# Identification of Neuroendocrine Stress Response-Related Circulating MicroRNAs as Biomarkers for Type 2 Diabetes Mellitus and Insulin Resistance

**DOI:** 10.3389/fendo.2018.00132

**Published:** 2018-03-28

**Authors:** Ying-Zhi Liang, Jing Dong, Jie Zhang, Shuo Wang, Yan He, Yu-Xiang Yan

**Affiliations:** ^1^Department of Epidemiology and Biostatistics, School of Public Health, Capital Medical University, Beijing, China; ^2^Municipal Key Laboratory of Clinical Epidemiology, Beijing, China; ^3^Health Medical Examination Center, Xuanwu Hospital, Capital Medical University, Beijing, China

**Keywords:** neuroendocrine, stress, microRNA, type 2 diabetes mellitus, insulin resistance, biomarker

## Abstract

**Background:**

Chronic stress plays an important role in the development of type 2 diabetes mellitus (T2DM) and insulin resistance (IR). MicroRNAs (miRNAs) play key roles in mediating stress responses by regulating the expression of target genes. This study systematically screened and identified the neuroendocrine stress response-related circulating miRNAs which are associated with T2DM and IR.

**Methods:**

Based on the differential plasma expression profiles between individuals with and without T2DM, stress-related miRNAs were selected from those differently expressed miRNAs whose targets are involved in known neuroendocrine pathway of stress response. Candidate miRNAs were further validated by quantitative real-time polymerase chain reaction in a large sample, including 112 T2DM patients, 72 individuals with impaired fasting glucose (IFG), and 94 healthy controls. The association between miRNA expression and potential risk of T2DM and IFG was assessed by multivariate logistic regression models. The miRNA predictors of IR were identified by stepwise multiple regression analysis. The diagnostic performance for T2DM was evaluated by area under the curve (AUC) of receiver operating characteristic (ROC).

**Results:**

let-7b, let-7i, miR-142, miR-144, miR-155, and miR-29a were selected as candidate miRNAs for validation. Increased expression of let-7b, miR-144, and miR-29a and decreased expression of miR-142 were significant independent predictors of T2DM, IFG, and IR (*P* < 0.0125). These miRNAs significantly correlated with stress hormone levels (*P* < 0.0125). A three-miRNA panel, including let-7b, miR-142, and miR-144 had a high accuracy for diagnosing T2DM (AUC = 0.871, 95% CI: 0.822–0.919).

**Conclusion:**

let-7b, miR-142, miR-144, and miR-29a in plasma may be important markers of neuroendocrine stress response and may play a role in the pathogenesis of T2DM and IR.

## Introduction

Type 2 diabetes mellitus (T2DM) and its complications have been identified as a major public health problem with a great impact on global morbidity and mortality, and heavy economic burden in both developed and developing countries (1). In 2015, 415 million people had diabetes and the number is predicted to increase to 642 million by 2040 (2). With increasing urbanization and strong economic growth in modern society, psychosocial stress has now emerged as an important contributor to the development of metabolic disorders. Chronic stress has been shown to cause impaired glucose homeostasis in earlier experimental and human researches (3). Recent epidemiological studies have confirmed the association between psychological stress and increasing risk of insulin resistance (IR), central obesity, and T2DM (4–6).

Persistent psychological stress can affect health through neuroendocrine pathways including hypothalamus-pituitary-adrenal (HPA) axis and sympathetic nervous system (SNS). The catecholamines epinephrine (E) and norepinephrine (NE), produced by the SNS, and cortisol, produced by the HPA axis, are the major stress hormones and important mediators between psychological stress and impaired glucose and lipid metabolism. Several researches have shown that increased activation of HPA axis and SNS, and consequent elevation of hormones might be responsible for the etiology and development of T2DM (7, 8).

MicroRNAs (miRNAs) are small non-coding regulatory RNAs which have proven powerful tools in finding disease associated pathways. They primarily regulate the expression of target genes at the post-transcriptional level by inhibiting translation or causing degradation of the target mRNA (9). Emerging data suggest that stress conditions can alter the biogenesis of miRNAs, which in turn determines cellular processes in response to stresses (10). Acute and chronic immobilization stress differentially altered the expression of miR-183 and miR-134 in stress-responsive regions of the rat brain (11). Both miR-134 and miR-183 were upregulated under acute stress in the central amygdala, while miR-134 showed decreasing expression with chronic stress in both the central amygdala and the hippocampus CA1 region. Increased serum miR-29c expression was identified after an acute social stress task in healthy participants (12). Levels of seven miRNAs (miR-16, -20b, -26b, -29a, -126, -144-5p, and -144-3p) in peripheral blood were significantly elevated in association with significant downregulation of their target mRNAs with chronic academic stress (13). Previous investigation in healthy occupational population showed that psychological stress increases plasma cortisol, which subsequently impacts expression of glucocorticoid receptor (GR) in peripheral blood mononuclear cells (14). Based on the above evidences, we assume that miRNAs may involve neuroendocrine pathway of stress responses and contribute to the susceptibility of T2DM.

In addition to their typical intracellular function, miRNAs also participate in cell-to-cell communication by actively secreting into the extracellular media, including serum, plasma, and small lipid vesicles, such as microvescicles and/or exosomes (15–17). Circulating miRNAs found in blood change with the physiological condition of the organism and may help to predict, diagnose, and follow-up metabolic disease (18). Therefore, circulating miRNAs may serve as valuable biomarkers and providing therapeutic benefits for T2DM.

In this study, it is the first time that the association between neuroendocrine stress response-related circulating miRNAs and T2DM was explored in an occupational population. The reason for our choice of such a population was that work environment issues are considered to be one of the most important psychosocial stress in contemporary societies, especially in urban China (14, 19). Based on an integrative analysis of differential miRNA expression profiles between T2DM patients and matched control subjects and target prediction, six candidate miRNAs that may involve in regulation of neuroendocrine stress response were selected. To reveal the role of these miRNAs in the development of T2DM, we also examined their expression changes in prediabetes and their association with insulin resistance.

## Materials and Methods

### Study Design

This study was performed based on a three-stage design. First, we compared the plasma miRNA profiles of individuals from an occupational population with and without T2DM. Neuroendocrine stress response-related miRNAs were then selected from those differently expressed miRNAs whose targets are involved in known biological pathways of stress response, including HPA axis and SNS. A large sample was eventually used to validate the expression of candidate miRNAs by quantitative real-time polymerase chain reaction (*q*RT-PCR).

### Subjects

The subjects in this study were Han Chinese individuals from a functional community cohort which was established in 2010 (20). This cohort was composed of nearly 9,000 employees from 64 companies (including white-collar and blue-collar workers from governments, schools, hospitals, factories, business, and service institutions) in Xuanwu district, representing most of the occupational population in urban Beijing. All the individuals in the cohort took annual physical examination at the health medical examination center of Beijing Xuanwu Hospital, Capital Medical University. The inclusion and exclusion criteria of the sample were described previously (20).

For the first stage, 10 T2DM patients and 10 individual matched controls (gender and age difference <12 months) were selected from the cohort in 2015 to compare the expression profile of miRNAs. At the validation stage, all the 112 newly diagnosed T2DM cases aged from 30–65 years old during 2017 were recruited. 94 health individuals with normal fasting plasma glucose (FPG < 6.1 mmol/l) were selected as frequency matched controls according to gender and age (±3 years). Another 72 individuals with impaired fasting glucose (IFG) were also recruited as prediabetes cases. The 1999 World Health Organization diagnostic criteria were used to diagnose T2DM (fasting glucose ≥ 7.0 mmol/l and/or 2 h glucose ≥ 11.1 mmol/l) and IFG (fasting glucose ≥ 6.1 and <7.0 mmol/l).

The exclusion criteria were as follows: (1) a past history of T2DM and using antidiabetic drugs according to the medical records, (2) any clinically acute or chronic inflammatory diseases, (3) liver and kidney dysfunction, (4) severe heart diseases, (5) mental illness, (6) gastrointestinal diseases, (7) any endocrine disease other than T2DM, (8) psychiatric disorders.

Each participant signed an informed consent form and this study was approved by the Ethical Committee of Capital Medical University.

### Data Collection and Anthropometric Measurement

Information about demographic data, medical history, current medication, and environmental exposure history were collected by a structured questionnaire (21). Anthropometric parameters, including weight, height, waist circumference (WC), and blood pressure were obtained using standard measurement described previously (6). Body mass index (BMI) was calculated as weight (kg) divided by height squared (m^2^).

### Plasma Collection

Following an overnight fast, peripheral blood (5 ml) was collected using EDTA-containing tubes between 7:30 and 8:30 a.m. Samples were immediately centrifuged at 2,000 r/s for 10 min at 4°C, generating the one-step centrifugation plasma samples. Then, the upper plasma phase was transferred to 1.5 ml tubes and centrifuged at 13,000 r/s for another 10 min at 4°C to remove additional cellular debris and minimize contamination of cell-free nucleic acids (gDNA and RNA) from damaged blood cells. The two-step centrifugated plasma samples were then stored at −80°C until further analysis.

### Biochemical Analysis

Fast plasma glucose, triglycerides (TG), total cholesterol (TC), high-density lipoprotein cholesterol (HDLC), and low-density lipoprotein cholesterol (LDLC) were tested through standard laboratory methods (Hitachi autoanalyzer 7060, Hitachi, Japan). Glycated hemoglobin was evaluated by high-pressure liquid chromatography method (Tosoh Corporation, Japan).

Plasma adrenaline (E), norepinephrine (NE), cortisol, and insulin were measured by commercial radioimmunoassays using with a γ counter (XH-6020, North Institute of Bio-Tech, China). Plasma corticotropin-releasing hormone (CRH), interleukin-6 (IL-6) concentrations were determined by enzyme linked immunosorbent assay using microplate reader (STAT FAX 2100, Awareness, USA). The coefficient of variation of these assays is <6.0% for the intra-assay and <10.0% for the inter-assay, respectively. The degree of insulin resistance was evaluated using the homeostasis model assessment of insulin resistance (HOMA-IR) calculated as: [fasting insulin (μIU/ml) × fasting glucose (mmol/l)]/22.5.

### RNA Extraction

Total RNA, including miRNA, was isolated from plasma using Trizol LS according to the manufacturer’s protocol (Invitrogen, USA). The concentration and purity of RNA was determined by NanoDrop 2000 spectrophotometer (Thermo Scientific, USA). Agarose gel electrophoresis stained with ethidium bromide was used to evaluate the integrity of RNA. All RNA used had A260/280 ratio >1.8 and electrophoresis showed integrity is acceptable.

### miRNA Microarray Expression Profiling

100 ng of total RNA from each plasma sample was labeled and hybridized on human Agilent miRNA microarrays (8*60 K, Design ID: 046064) using the miRNA Labeling Reagent and Hybridization Kit (Agilent Technologies, USA) following the manufacturer’s protocol. Briefly, total RNA was dephosphorylated, denatured, and then labeled with Cy3-CTP. After purification, the labeled RNAs were hybridized onto the microarray. The hybridized array was then washed and scanned using high dynamic range settings according to Agilent specifications and data was extracted from the scanned image using Feature Extraction version 10.2 (Agilent Technologies). The miRNA array data were analyzed for data summarization, normalization, and quality control by GeneSpring GX 12.1 Software (Agilent Technologies). To select the differentially expressed genes, we used threshold values of 2-fold change (FC) and a paired *t*-test *P* value of 0.05.

### Selection of Neuroendocrine Stress Response-Related Candidate miRNAs

Among the differently expressed miRNAs from microarray analyze, those involved in biological pathways of stress response, including HAP axis and SNS, were selected for validating their association with T2DM in a larger sample. The criteria of candidate miRNAs, including: (1) the target genes are important regulators of stress-induced activation, including hormone (CRH, COR, N, NE) secreting and hormone receptors; (2) the target genes should be identified by published literature (PubMed database) or at least two prediction programs, including miRecords (2013), TarBase (v 7.0), and miRTarBase (2015).

### Real-Time Quantitative PCR

Quantification of miRNAs was performed with a two-step reaction process: reverse transcription (RT) and PCR. cDNA synthesis was carried out using the miScript Reverse Transcriptase Kit (Qiagen, Germany) according to manufacturer’s instructions with a GeneAmp^®^ PCR System 9700 (Applied Biosystems, USA). Briefly, each RT reaction contained 1 µg RNA, 4 µl of miScript HiSpec Buffer, 2 µl of Nucleics Mix, and 2 µl of miScript Reverse Transcriptase Mix. The 20 µl reactions were incubated for 60 min at 37°C, 5 min at 95°C, and then held at 4°C. The PCR reactions were performed using 1 µl of the first strand cDNA on LightCycler^®^ 480 II Real-time PCR Instrument (Roche, Swiss) in total volume of 10 µl with 5 µl of 2 × LightCycler^®^ 480 SYBR Green I Master (Roche, Swiss), 0.2 µl of universal primer (Qiagen, Germany), 0.2 µl of microRNA-specific primer, and 3.6 µl of nuclease-free water. The reactions were carried out in a 386-well plate at 95°C for 10 min, followed by 40 cycles of 95°C for 15 sec, and 60°C for 30 sec. Each sample was analyzed in triplicate.

A melting curve analysis was performed at the end of the PCR cycle to validate the specific generation of the expected PCR product. The miRNA-specific primer sequences were designed in the laboratory and synthesized by Generay Biotech (Generay, PRC) based on the miRNA sequences obtained from the miRBase database (Release 20.0).

We used miR-451a as an internal control due to its stable and consistent expression throughout all the evaluated samples. Triplicate values of each sample were normalized to miR-451a. The miRNA relative expression values were calculated as 2^−ΔCt^, [ΔCt = mean Ct (miR-X)—mean Ct (miR-451a)].

### Statistical Analysis

The data were assessed for normal distribution with the Kolmogorov–Smirnov test and were logarithmically transformed for statistical analyses when necessary. Chi-square test or one-way analysis of variance followed by least significant difference test was used to examine the differences between the groups of subjects. Those significant differently expressed miRNAs among the three groups were used in further analysis. The associations between miRNA expression levels and risk of T2DM and IFG were assessed by using both univariate and multivariate logistic regression models with or without adjustment for potential confounders. The relationship between miRNA markers and stress hormone and HOMA-IR were evaluated by spearman’s correlation coefficient. The miRNA predictors of IR were further identified by stepwise multiple regression. The area under the curve (AUC) of receiver operating characteristic (ROC) was used as diagnostic index for evaluating the biomarker potential of each miRNA and miRNA panel for T2DM. A *P* value of less than 0.05 was considered statistical significance. All statistical analyses were conducted with SPSS software, version 21.0 (IBM SPSS, Inc., Chicago, IL, USA). The graphs were designed by GraphPad Prism 5.01 (Graphpad Software Inc., San Diego, CA, USA).

## Results

### Identification of Altered miRNA Expression Associated With Chronic Stress Response

**Figure 1:**
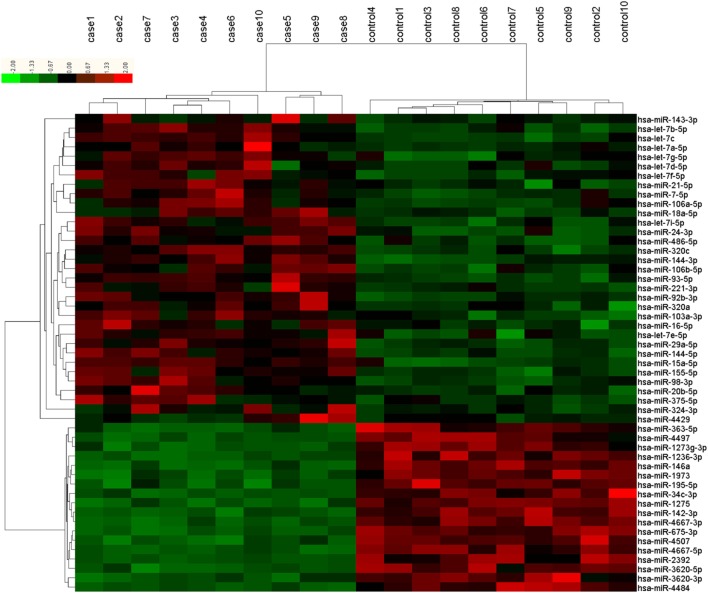
Differentially expressed plasma microRNAs (miRNAs) between type 2 diabetes mellitus (T2DM) patients (*n* = 10) and healthy controls (*n* = 10) in microarray. Each row represents one miRNA, and each column represents a plasma sample. The legend on the right indicates the miRNA represented in the corresponding row. The relative miRNA expression is depicted according to the color scale. Red indicates upregulation; green indicates downregulation. The numbers with case indicate newly diagnosed T2DM patients; numbers with control indicate healthy controls.

The microarray analysis identified 51 human mature miRNAs that differentially expressed in plasma of 10 T2DM patients and 10 controls (Table 1) by using absolute value of log_2_FC > 1 and *P* < 0.05 as cutoff values, including 33 upregulated miRNAs and 18 downregulated miRNAs (Table S1 in Supplementary Material). Hierarchical clustering of miRNA expression patterns correctly classified the two groups (Figure 1). Six miRNAs involved in pathways of chronic stress response, as determined by target gene prediction and literature analysis, were further selected as candidate miRNAs for validation study with a larger sample. These miRNAs are let-7b, let-7i, miR-142, miR-144, miR-155, and miR-29a. The six miRNAs that could significantly discriminate the T2DM from controls and other related details, including the potential target genes were summarized in Table 2.

**Table 1:** Demographic and clinical characteristics of participants in the microRNA profile study.

Variable	Type 2 diabetes mellitus (*n* = 10)	Control (*n* = 10)	*P*
Age (years)	49.10 ± 3.84	49.00 ± 3.97	0.955
Gender (male/female)	5/5	5/5	1.000*
BMI (kg/m^2^)	26.84 ± 2.85	22.74 ± 1.91	0.001
WC (cm)	89.74 ± 11.78	78.80 ± 6.66	0.020
SBP (mmHg)	125.50 ± 12.98	114.20 ± 18.04	0.125
DBP (mmHg)	79.30 ± 7.07	73.00 ± 6.72	0.056
TC (mmol/l)	5.49 ± 1.14	4.87 ± 0.83	0.184
TG (mmol/l)	2.28 ± 2.12	1.02 ± 0.39	0.094
LDLC (mmol/l)	2.79 ± 0.84	2.35 ± 0.43	0.166
HDLC (mmol/l)	1.38 ± 0.43	1.83 ± 0.40	0.028
FPG (mmol/l)	11.91 ± 1.83	4.93 ± 0.40	<0.001
HbA1c (%)	8.66 ± 0.87	4.97 ± 0.24	<0.001
Insulin (uIU/ml)	16.50 ± 3.49	10.58 ± 1.52	<0.001
HOMA-IR	8.64 ± 2.32	2.32 ± 0.43	<0.001
Smoking (*n*)	0	0	–
Alcohol use (*n*)	0	1	0.500*
Physical activity (*n*)	7	5	0.650*

*BMI, body mass index; WC, waist circumference; SBP, systolic blood pressure; DBP, diastolic blood pressure; TC, total cholesterol; TG, triglyceride; LDLC, low-density lipoprotein cholesterol; HDLC, high-density lipoprotein cholesterol; FPG, fast plasma glucose; HOMA-IR, homeostasis model assessment of insulin*.

*Fisher exact P value.

**Table 2:** Differential microRNAs (miRNA) expression in type 2 diabetes mellitus (T2DM) vs. control group.

miRNA	Mean intensities in T2DM	Mean intensities in controls	*P* value	Fold change	Up/Down	(Predicted) target gene	Prediction programs
let-7b-5p	1,305	397	2.79E-07	3.28	Up	ADRB2/ADRB3 (22)	a,b
let-7i-5p	461	173	2.41E-09	2.66	Up	ADRB2/ADRB3	a,b
miR-142-3p	503	1,040	5.47E-06	0.48	Down	Corticotropin-releasing hormone (CRH)/interleukin-6 (23)	a,b,c
miR-144-3p	219	107	6.68E-11	2.05	Up	NR3C1 (24)	a,c
miR-155-5p	204	100	4.56E-09	2.04	Up	NR3C1/CYP11B1	a,b,c
miR-29a-5p	208	98	7.73E-09	2.12	Up	NR3C1/CRHR1	a,b

*a, miRecords; b, TarBase; c, miRTarBase*.

### Basic Characteristics of the Participants in the Validation Study

The demographic and clinical characteristics of the participants were presented according to the study groups (Table 3). There was no significant difference in the distribution of age, gender, smoking, alcohol use, and physical activity among three groups (*P* > 0.05). Most of the clinical parameters, including BMI, WC, SBP, DBP, LDLC, PFG, insulin, HbA1c, and HOMA-IR showed significant higher levels in T2DM and IFG groups than those in controls (*P* < 0.05). Even compared to the IFG group, T2DM group also showed significant higher levels of WC, DBP, LDLC, PFG, insulin, HbA1c, and HOMA-IR (*P* < 0.05).

**Table 3:** Demographic and clinical characteristics of study participants in the validation study.

Variable	Type 2 diabetes mellitus (*n* = 112)	Impaired fasting glucose (IFG) (*n* = 72)	Control (*n* = 94)	*P*
Age (years)	54.75 ± 7.53	52.53 ± 7.51	52.84 ± 8.85	0.111
Gender (male/female)	69/43	38/34	53/41	0.478*
BMI (kg/m^2^)	27.11 ± 3.17^a^	26.26 ± 2.97^a^	23.86 ± 3.27	<0.001
WC(cm)	92.12 ± 8.46^a,b^	88.93 ± 9.18^a^	82.05 ± 9.57	<0.001
SBP (mmHg)	138.82 ± 21.19^a^	133.98 ± 17.57^a^	123.90 ± 17.85	<0.001
DBP (mmHg)	86.30 ± 12.64^a,b^	82.39 ± 10.53^a^	77.98 ± 12.33	<0.001
TC(mmol/l)	5.49 ± 1.15^a,b^	5.07 ± 0.93	4.91 ± 0.94	<0.001
TG(mmol/l)	2.98 ± 2.86^a,b^	1.88 ± 1.04	1.71 ± 1.43	<0.001
LDLC (mmol/l)	3.26 ± 1.25^a,b^	2.81 ± 0.81^a^	2.40 ± 0.82	<0.001
HDLC (mmol/l)	1.21 ± 0.57^a^	1.29 ± 0.34	1.41 ± 0.39	0.008
FPG (mmol/l)	9.48 ± 2.62^a,b^	6.44 ± 0.24^a^	4.94 ± 0.38	<0.001
HbA1c (%)	7.58 ± 1.54^a,b^	5.74 ± 0.60^a^	5.16 ± 0.39	<0.001
Insulin (uIU/ml)	14.88 ± 2.95^a,b^	13.05 ± 2.67^a^	9.27 ± 2.55	<0.001
HOMA-IR	6.31 ± 2.19^a,b^	3.74 ± 0.82^a^	2.04 ± 0.59	<0.001
Smoking (*n*, %)	16, 17.02	11, 15.28	17, 15.18	0.750*
Alcohol use (*n*, %)	12, 10.71	9, 12.50^a^	5, 5.32	0.236*
Physical activity (*n*, %)	71, 63.39	47, 65.28	69, 73.40	0.286*

*BMI, body mass index; WC, waist circumference; SBP, systolic blood pressure; DBP, diastolic blood pressure; TC, total cholesterol; TG, triglyceride; LDLC, low-density lipoprotein cholesterol; HDLC, high-density lipoprotein cholesterol; FPG, fast plasma glucose; HOMA-IR, homeostasis model assessment of insulin*.

*χ^2^ value.

*^a^Significantly different from control group (*P* < 0.05)*.

*^b^Significantly different from IFG group (*P* < 0.05)*.

### Comparison of Stress-Related miRNA and Hormone Levels Among T2DM, IFG, and Control Groups

**Figure 2:**
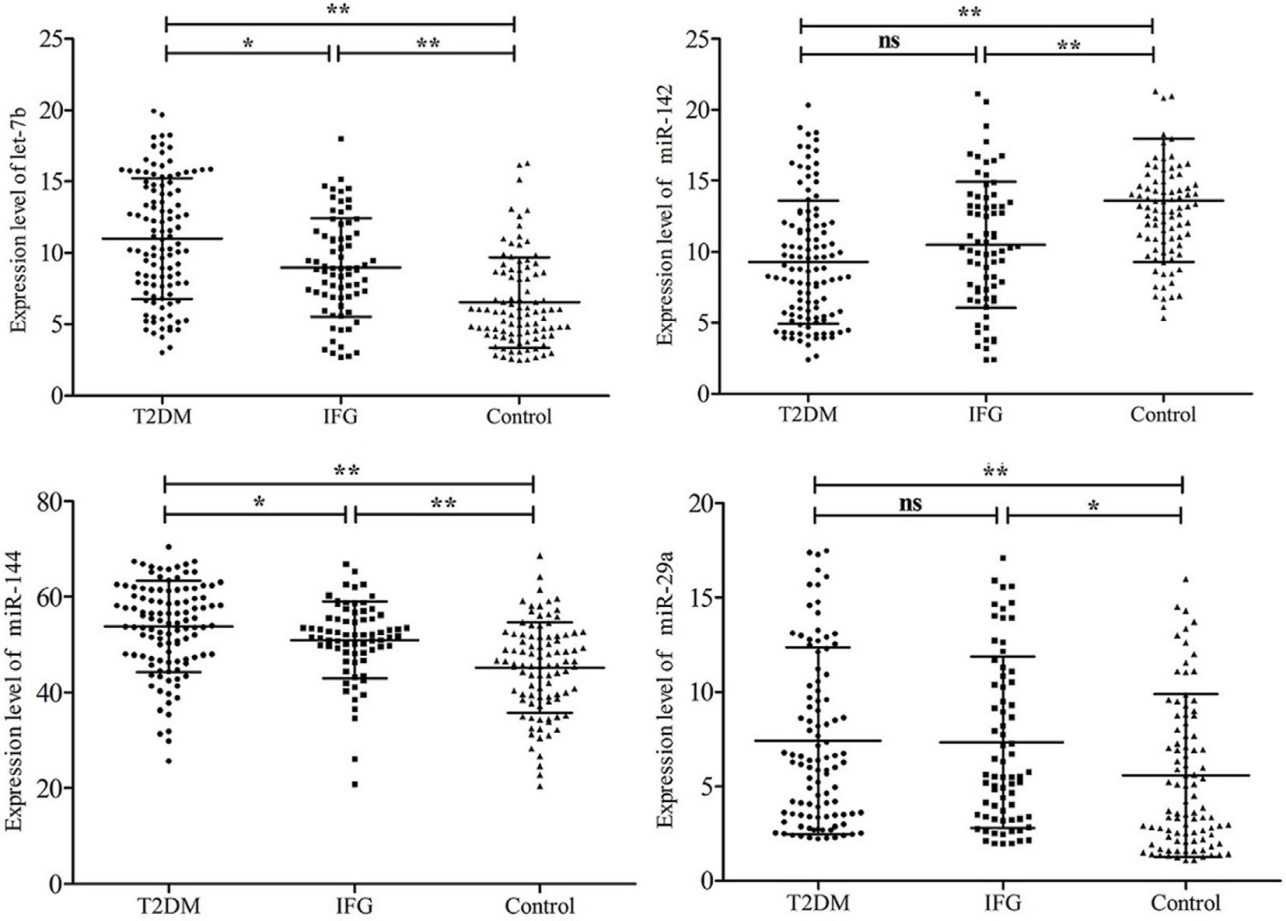
Comparison of plasma microRNAs expression based on quantitative real-time polymerase chain reaction readings among type 2 diabetes mellitus, impaired fasting glucose, and control groups. Data are represented by scatter diagram. *P < 0.05, **P < 0.01, ns, not significant.

The plasma levels of the six stress-related candidate miRNAs measured by *q*RT-PCR were listed in Table 4. There are four miRNAs, including let-7b, miR-142, miR-144, and miR-29a showed significant different expression levels among the three groups (*P* < 0.05). Compared with control group, expression of let-7b, miR-144, and miR-29a in T2DM group and expression of let-7b and miR-144 in IFG group were significantly upregulated (*P* < 0.05) (Figure 2). By contrast, expression of miR-142 in subjects with T2DM and IFG were significantly downregulated when compared with control group (*P* < 0.001). However, there was no significant difference between T2DM and IFG groups for miR-29a (*P* = 0.919) and miR-142 (*P* = 0.064) (Figure 2). As the potential biomarkers of T2DM, the predictive and clinical significance of let-7b, miR-142, miR-144, and miR-29a were evaluated in the further statistical analyses.

**Table 4:** MicroRNA and stress hormonal characteristics in the three compared groups.

Variable	Type 2 diabetes mellitus (*n* = 112)	Impaired fasting glucose (IFG) (*n* = 72)	Control (*n* = 94)	*P*
let-7b	11.00 ± 4.23^a,b^	8.98 ± 3.44^a^	6.52 ± 3.15	<0.001
let-7i	7.08 ± 4.07	6.82 ± 4.19	6.48 ± 4.39	0.314
miR-142	9.25 ± 4.34^a^	10.48 ± 4.44^a^	13.61 ± 4.35	<0.001
miR-144	53.82 ± 9.54^a,b^	50.98 ± 8.02^a^	45.20 ± 9.51	<0.001
miR-155	0.59 ± 0.73	0.61 ± 0.67	0.63 ± 0.80	0.940*
miR-29a	7.42 ± 4.95^a^	7.35 ± 4.55^a^	5.58 ± 4.31	0.001
E (pg/ml)	153.34 ± 49.91^a^	139.09 ± 48.60	135.80 ± 52.98	0.032
NE (pg/ml)	413.00 ± 94.57^a^	392.20 ± 88.04	379.40 ± 102.26	0.041
Cortisol (ng/ml)	219.62 ± 36.59^a,b^	204.50 ± 35.22^a^	176.49 ± 34.74	<0.001
CRH (ng/ml)	5.40 ± 0.61^a^	5.39 ± 0.55^a^	5.04 ± 0.47	<0.001
IL-6 (pg/ml)	134.07 ± 16.62^a,b^	126.58 ± 19.26	122.50 ± 21.51	<0.001

*E, adrenaline; NE, norepinephrine; CRH, corticotropin-releasing hormone; IL-6, interleukin-6*.

*Skewed distributed and analyzed by log-transformed values.

*^a^Significantly different from control group (*P* < 0.05)*.

*^b^Significantly different from IFG group (*P* < 0.05)*.

As presented in Table 4, the plasma levels of E, NE, Cortisol, CRH, and IL-6 significantly higher in T2DM group than those in control group (*P* < 0.05). Cortisol and CRH also showed significantly higher levels in the IFG group when compared with controls (*P* < 0.05).

### The Relationship Between miRNA Expression and Stress Hormones

The spearman’s correlation analysis revealed that plasma stress hormones are significantly associated with expression of let-7b, miR-142, miR-144, and miR-29a in the study subjects at the multiple test corrected threshold of 0.0125 (assuming family wise a level of 0.05 and number of miRNA markers) (Table 5). The partial correlation between miRNAs and stress hormones were further analyzed by correction for FBG and HOMA-IR. The significant correlations were consistent with bivariate correlations. However, when data was stratified according to the FBG level, no significant correlation between miRNAs and hormones were identified in the subgroup analysis (*P* > 0.0125).

**Table 5:** Spearman correlation among the microRNAs and stress hormones and HOMA-IR.

	E	NE	Cortisol	CRH	IL-6	HOMA-IR
**Total subjects**						
let-7b	0.213*	0.216*	0.324*	0.257*	0.164*	0.469*
miR-142	−0.086	−0.102	−0.241*	−0.304*	−0.225*	−0.436*
miR-144	0.063	0.086	0.343*	0.198*	0.139	0.419*
miR-29a	−0.029	−0.025	0.183*	0.120	0.123	0.196*
**Type 2 diabetes mellitus group**						
let-7b	0.213	0.191	0.105	0.207	0.095	0.214
miR-142	−0.053	−0.069	−0.068	−0.200	−0.221	−0.223
miR-144	−0.023	−0.017	0.226	0.174	0.025	0.202
miR-29a	−0.114	−0.123	0.062	−0.003	0.110	0.149
**Impaired fasting glucose group**						
let-7b	0.187	0.180	0.011	0.092	0.028	0.165
miR-142	−0.046	−0.042	−0.110	−0.173	−0.177	−0.184
miR-144	0.147	0.166	0.192	−0.013	0.046	0.192
miR-29a	−0.077	−0.078	0.117	0.033	0.120	0.045
**Control group**						
let-7b	0.092	0.101	0.179	0.129	0.082	0.030
miR-142	0.026	0.001	0.095	−0.157	−0.079	−0.014
miR-144	−0.077	−0.037	0.145	0.077	0.045	0.092
miR-29a	0.037	0.033	0.119	0.115	0.022	0.019

*E, adrenaline; NE, norepinephrine; CRH, corticotropin-releasing hormone; IL-6, interleukin-6; HOMA-IR, homeostasis model assessment of insulin*.

*P < 0.0125.

There are some significant associations existed among the stress hormones, including E and NE (*r* = 0.982, *P* < 0.001), cortisol and CRH (*r* = 0.265, *P* < 0.001), and IL-6 and cortisol (*r* = 0.159, *P* = 0.008) in the study subjects.

### Association of miRNA Expression With Risk of T2DM and IFG

Univariate and multivariate logistic regression revealed that altered levels of let-7b, miR-142, miR-144, and miR-29a were significantly associated with T2DM and IFG, even after adjustment for possible confounding variables (*P* < 0.05) (Table 6). Upregulated expression of let-7b, miR-144, and miR-29a and downregulated expression of miR-142 were predictors of increased risk of T2DM and IFG, respectively. Among the four miRNAs, let-7b showed the highest level of association with T2DM (Adjusted OR = 1.33, 95% CI 1.10–1.50, *P* < 0.001) and IFG (Adjusted OR = 1.20, 95% CI 1.07–1.35, *P* = 0.002).

**Table 6:** Univariate and multiple logistic regression analysis for the risk of type 2 diabetes mellitus (T2DM) and impaired fasting glucose (IFG).

Models	T2DM		IFG	
	OR (95% CI)	*P* value	OR* (95% CI)	*P* value
**let-7b**				
Univariate model	1.36 (1.24, 1.49)	<0.001	1.25 (1.23, 1.38)	<0.001
Multivariate model 1^a^	1.36 (1.23, 1.49)	<0.001	1.22 (1.10, 1.35)	<0.001
Multivariate model 2^b^	1.35 (1.22, 1.50)	<0.001	1.23 (1.10, 1.37)	<0.001
Multivariate model 3^c^	1.33 (1.10, 1.50)	<0.001	1.20 (1.07, 1.35)	0.002
**miR-142**				
Univariate model	0.80 (0.74, 0.86)	<0.001	0.85 (0.78, 0.92)	<0.001
Multivariate model 1^a^	0.78 (0.73, 0.85)	<0.001	0.84 (0.77, 0.92)	<0.001
Multivariate model 2^b^	0.80 (0.74, 0.88)	<0.001	0.86 (0.79, 0.95)	0.001
Multivariate model 3^c^	0.83 (0.75, 0.93)	<0.001	0.86 (0.78, 0.94)	0.002
**miR-144**				
Univariate model	1.10 (1.06, 1.14)	<0.001	1.08 (1.04, 1.12)	<0.001
Multivariate model 1^a^	1.10 (1.06, 1.14)	<0.001	1.12 (1.07, 1.18)	<0.001
Multivariate model 2^b^	1.09 (1.05, 1.13)	<0.001	1.11 (1.05, 1.17)	<0.001
Multivariate model 3^c^	1.08 (1.03, 1.13)	0.004	1.12 (1.05, 1.18)	<0.001
**miR-29a**				
Univariate model	1.09 (1.03, 1.16)	0.007	1.09 (1.02, 1.18)	0.014
Multivariate model 1^a^	1.09 (1.02, 1.14)	0.010	1.09 (1.01, 1.17)	0.025
Multivariate model 2^b^	1.08 (1.01, 1.16)	0.027	1.09 (1.01, 1.18)	0.027
Multivariate model 3^c^	1.10 (1.00, 1.20)	0.036	1.10 (1.01, 1.20)	0.033

*^a^Adjusted for age, gender, smoking, drinking, and physical activity*.

*^b^Further adjusted for BMI based on model 1*.

*^c^Further adjusted for total cholesterol, TG, HDLC, LDLC, SBP, and DBP based on model 2*.

*OR, odds ratio; CI, confidence interval*.

### Association of miRNA Expression With Insulin Resistance

Since insulin resistance is the core metabolic abnormality in T2DM, relationships between HOMA-IR and expression levels of miRNAs were further assessed. The spearman correlation analysis revealed that let-7b, miR-144 and miR-29a were significant positively related to HOMA-IR while miR-142 was negatively related to HOMA-IR in all subjects (Table 5). To confirm the association between miRNA and insulin resistance independent of obesity, multiple linear regression analysis was further performed with possible confounders were adjusted. As the results indicated, let-7b, miR-144 and miR-29a were significant positive predictors of HOMA-IR (*P* < 0.05) and miR-142 was a significant negative predictor of HOMA-IR (*P* < 0.05) independent of WC (Table 7). If WC was replaced by BMI among the above covariables in the linear regression model, these miRNAs were also independently associated with HOMA-IR (Table 7).

**Table 7:** Stepwise multiple linear regression analysis of the relationship between miRNA and homeostasis model assessment of insulin resistance.

Variables	Adjusted for BMI		Adjusted for WC	
	β coefficient	*P*	β coefficient	*P*
BMI/WC	0.106	0.004	0.034	0.009
Triglycerides (TG)	0.146	0.029	0.141	0.035
let-7b	0.142	<0.001	0.144	<0.001
miR-142	−0.068	0.010	−0.068	0.010
miR-144	0.045	<0.001	0.045	<0.001
miR-29a	0.049	0.043	0.049	0.047

*Variables entered in step 1: age, gender, smoking, drinking, physical activity, TCH, TG, HDLC, LDLC, SBP, DBP, WC/BMI, let-7b, miR-142, miR-144, and miR-29a*.

*WC, waist circumference; BMI, body mass index; HDLC, high-density lipoprotein cholesterol*.

### The Diagnostic Performance of the miRNAs

**Figure 3:**
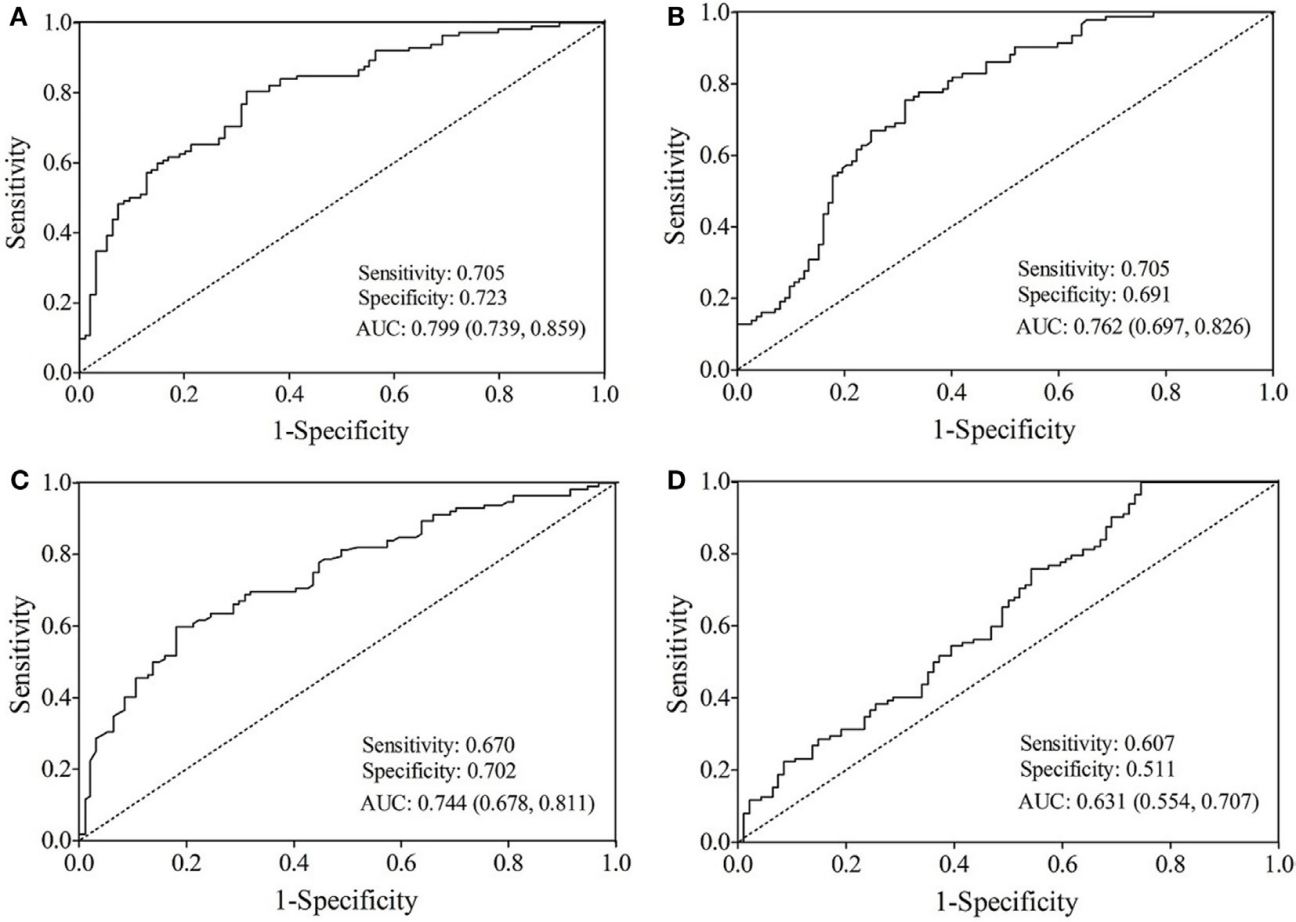
Receiver operating characteristic curve analysis for type 2 diabetes mellitus prediction. Area under the curve (AUC) estimation for the microRNAs: **(A)** let-7b, **(B)** miR-142, **(C)** miR-144, and **(D)** miR-29a.

**Figure 4:**
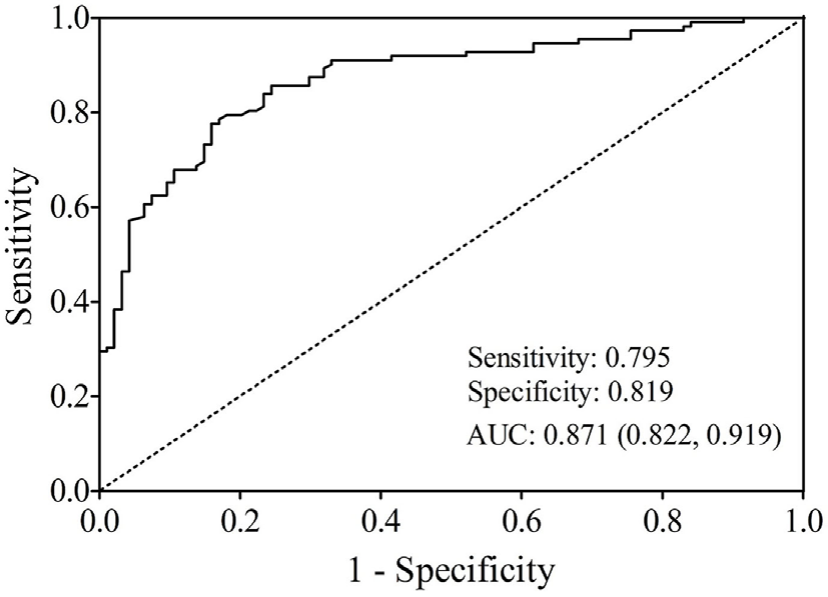
Receiver operating characteristic plot for the miRNA panel (let-7b, miR-142, and miR-144) discriminating type 2 diabetes mellitus.

The AUC value of let-7b, miR-142, miR-144, and miR-29a for diagnosing T2DM was 0.799 (cutoff value: 8.29), 0.762 (cutoff value: 11.39), 0.744 (cutoff value: 50.73), and 0.631 (cutoff value: 4.19), respectively (Figure 3). By using stepwise logistic regression model, a three-miRNA panel, including let-7b, miR-142, and miR-144 were identified for diagnosing T2DM (*P* < 0.05). The predicted probability of T2DM based on the miRNA panel, logit (*P* = T2DM) = −3.916 + 0.229 × let-7b—0.160 × miR-142 + 0.073 × miR-144 was used as surrogate maker to construct the ROC curve. The AUC for the established miRNA panel was 0.871 (95% CI: 0.822–0.919, Figure 4). These results demonstrated that the miRNA panel had high accuracy in discriminating T2DM from healthy controls (*P* < 0.001).

## Discussion

This study identified the association between plasma expression of neuroendocrine stress response-related miRNAs and T2DM as well as IR in an occupational cohort. Based on the differential miRNA expression profile and bioinformatic analysis, six stress-related miRNAs (let-7b, let-7i, miR-142, miR-144, miR-155, and miR-29a) associated with T2DM were selected for *q*RT-PCR validation. We found that the average expression levels of let-7b, miR-142, miR-144, and miR-29a were significantly different between T2DM patients and healthy controls. The increased expression of let-7b, miR-144, and miR-29a and decreased expression of miR-142 were significant-independent predictors of T2DM, IFG, and even IR. At the same time, ROC analysis showed that a three-miRNA panel, including let-7b, miR-142, and miR-144 had a high accuracy for diagnosing T2DM.

A number of miRNAs have been shown to be important signatures or therapeutic targets in patients with T2DM in circulation and specific tissues (25, 26). miR-184 was reported as a highly regulated miRNA impacting the growth and function of the β-cell. By targets Argonaute2, miR-184 plays adaptive role of the miRNA pathway based on metabolic state (27). Stress-induced overexpression of miR-708 suppressed β-cell proliferation and induced β-cell apoptosis, which provide a novel mechanism of glucose regulation of β-cell function and growth (28). Comparing to tissue biomarkers, circulating miRNAs are preferred due to easier sampling and testing. Circulating miR-192 and miR-193b were even found significantly increased in the prediabetic state (29). Some other miRNAs have also been identified as biomarkers for diabetic complications, such as plasma miR-152 and diabetic nephropathy, and miR-93 and diabetic retinopathy (30, 31). Therefore, circulating miRNAs may help to predict the development and progression of T2DM.

Increasing evidence indicates that psychosocial factors facilitate the development and progression of central obesity and T2DM, possibly as the results of increasing insulin resistance (32). In our previous study, the Copenhagen Psychosocial Questionnaire was used to assess job-related psychosocial stress. We found that after adjustment for the influence of cortisol, HOMA-IR was also significantly associated with scales of “demands at work” and “insecurity at work” (6). Psychosocial factors interact with biological mechanisms at several levels in the organisms include neuroendocrine, metabolic, and molecular responses. Stress hormones influence glucose metabolism by stimulating gluconeogenesis and impairing glucose uptake in peripheral tissues like fat and muscle (8, 33). Activation of stress hormones secretion is mediated by neuronal signals. Repeated stress activates hypothalamic paraventricular nucleus to synthesize CRH, the major regulator of stress responses. Anterior pituitary responds to CRH to produce adrenocorticotropic hormone, which is released into the circulation and promotes synthesis and release of glucocorticoids (cortisol in humans) at the level of the adrenal gland (34). The HPA axis controlling cortisol secretion and the SNS both originate in the hypothalamus and they are interlinked, and hyperactivity in one of them can activate the other (35).

Our study showed that plasma levels of E, NE, cortisol, and CRH significantly higher in T2DM group than those in control group (*P* < 0.05). Cortisol and CRH also showed significantly higher levels in the IFG group when compared with controls (*P* < 0.05). These results provide evidence for the association between chronic stress and T2DM based on an occupational population study. Elevated activities in both HPA axis and SNS, which were triggered by psychosocial factors have also been reported in individuals with the metabolic syndrome (36). Increased plasma IL-6 levels in T2DM patients and correlation of IL-6 with cortisol in this study maybe another evidence in the pathway between chronic stress and T2DM, since various psychological stressors alone can induce proinflammatory (36, 37).

Cellular responsiveness to stress hormone depends on the amount of corresponding receptor protein. Significant upregulated expression of let-7b and miR-144 in T2DM patients in our study may indicates downregulated expression of their target gene, including ADBR2/ADBR3 (β-adrenoreceptors) and NR3C1 (GR), respectively. Sheng, et al. verified that the protein expression of ADRB3 was regulated by direct binding of let-7b at 3′-UTR (22). In addition, they found that let-7b was negatively correlated with ADRB3 protein level and had a decreased expression in perivascular adipose tissue of hypertensive mice. Cortisol treatment reduced NR3C1 mRNA levels and increased miR-144 expression in hippocampus and sperm of mice (24). The evidence of NR3C1 as the target of miR-144 can also be obtained from next-generation sequencing (miRTarBase). Positive correlation between let-7b and E/NE, miR-144 and cortisol in the study subjects indicated decreased stress hormone receptor expression, which reflects the negative feedback mechanisms for controlling the magnitude of responses to stress (34). Our previous population-based study showed that chronic stress increase plasma cortisol, which subsequently decrease mRNA expression of GRα and increase of GRβ/GRα mRNA ratio in lymphocyte (14).

miR-142, which mediated the expression of CRH, showed lower levels in either T2DM or IFG individuals compared with controls. miR-142 also participates in the inflammatory response by regulating the production of IL-6 (23). Both plasma CRH and IL-6 were negatively associated with miR-142 expression in total subjects, which may be explained by the inhibitory effect of miRNA on target genes. Microarray analysis revealed the downregulation miR-142 in the peripheral blood of mice associated with pronounced enhanced oxidative stress (22).

miR-29a was reported to be involved in the glucocorticoids signaling pathway (24) and chronic academic stress excessive glucocorticoids reduce the expression of miR-29a (13). The association may partially be explained by receptor gene NR3C1 and CRHR1. Although miR-29a expression significantly associated with T2DM and HOMA-IR, relatively weak correlations exist only between miR-29a and cortisol (*r* = 0.183) in the total subjects. It is possible that miR-29a regulates glucose metabolism though other pathway.

It is the first time that we systematically investigated the relationship between plasma expression of neuroendocrine stress response-related miRNAs and T2DM at population level. The reasonable diagnostic accuracy of miRNA panel (let-7b, miR-142, and miR-144) indicates their clinical value in diagnosing T2DM. It is worth noting that significantly altered expression of let-7b, miR-142, miR-144, and miR-29a was found even at the stage of pre-diabetes in this study. These miRNAs were also identified as significant-independent predictors of insulin resistance which is an important component of the pathophysiological processes that underlies the development of T2DM. The findings of this study support the hypothesis that neuroendocrine stress response-related miRNAs may play a role in the pathogenesis of T2DM and insulin resistance by regulating those target genes which involve neuroendocrine pathways of stress responses, which unveils new targets for the prevention, prediction, and more personalized treatment of neuroendocrine stress-related disorders, including T2DM.

Our results are reliable based on the three-stage design. However, there are several limitations in our study. The association between neuroendocrine stress response-related miRNAs and T2DM could not be proved causality; follow-up studies are needed to confirm our findings. Experimental study is needed to clarify the role of these miRNAs in the molecular mechanisms of T2DM.

## Ethics Statement

This study was approved by the Ethical Committee of Capital Medical University.

## Author Contributions

Y-XY designed the study. Y-ZL and JD collected the data. Y-XY and Y-ZL conducted bioinformatical analyses and statistical analyses. Y-ZL, JZ, SW, and YH conducted the experiments. All authors interpreted the data and all authors contributed to writing. All authors have approved the final manuscript.

## Conflict of Interest Statement

The authors declare that the research was conducted in the absence of any commercial or financial relationships that could be construed as a potential conflict of interest.

## Supplementary Material

Table S1 Data of differentially expressed plasma microRNAs (miRNAs) between type 2 diabetes mellitus (T2DM) patients and healthy controls in microarray.
